# User Satisfaction With Assistive, Rehabilitation, and Training Technologies: Questionnaire Development and Content Validation Study

**DOI:** 10.2196/88182

**Published:** 2026-09-25

**Authors:** Oskar Cvjetičanin, Ivana Marić, Nejc Šarabon

**Affiliations:** 1 Faculty of Health Sciences, University of Primorska, Polje 42, Izola, 6310, Slovenia; 2 Andrej Marušič Institute, University of Primorska, Koper, Slovenia; 3 Laboratory for Motor Control and Motor Behavior, S2P, Science to Practice, Ljubljana, Slovenia; 4 Ludwig Boltzmann Institute for Rehabilitation Research, St. Pölten, Austria

**Keywords:** user satisfaction, human-centered design, user-centered design, assistive technology, rehabilitation technology, training technology, questionnaire development

## Abstract

**Background:**

According to human-centered design principles, user experience and user satisfaction are key aspects when developing or providing patients and clients with assistive, rehabilitation, or training technologies. Existing tools for assessing user experience and satisfaction are often either too narrow (developed for specific technologies) or too broad, failing to capture all relevant aspects of the technology being evaluated.

**Objective:**

The main objective of this study was to present the development process and content validation of a questionnaire that, following further validation steps, could be useful for researchers and developers in assessing the satisfaction of direct end users with assistive, rehabilitation, and training technologies.

**Methods:**

The development of the User Satisfaction Questionnaire for Assistive, Rehabilitation, and Training Technologies (U-ART-Q) followed a multistep process: (1) literature review, (2) domain definition and item generation, (3) expert consultation, (4) linguistic validation, and (5) content validation. Initially, 72 items were generated and organized into 7 domains. After expert review and linguistic validation, the questionnaire underwent content validation by 8 experts in physiotherapy, kinesiology, and ergonomics. Each item was rated for relevance using a 4-point Likert-type scale. The content validity ratio (CVR) and item-level content validity index (I-CVI) were calculated, and items below the accepted thresholds were removed or further revised.

**Results:**

Expert consultation resulted in the removal or merging of 20 items to improve clarity and reduce redundancy. Of the 52 items evaluated during content validation, 44 met the CVR and I-CVI criteria (CVR >0.75 and I-CVI >0.78). Eight items required additional review, and 2 items were ultimately removed. The final questionnaire comprises 10 initial screening questions and 50 content-valid items across 7 domains: design, comfort, effectiveness and usefulness, service, treatment or exercise, overall impressions, and safety. The overall average content validity index was 0.915, providing evidence of strong content validity. A macro-enabled Excel tool was developed to automate item selection and generate a PDF version of the questionnaire based on initial screening responses.

**Conclusions:**

The U-ART-Q is a newly developed instrument for assessing user satisfaction with assistive, rehabilitation, and training technologies. Further research will focus on psychometric evaluation, reliability testing, and multilingual validation to determine whether the U-ART-Q can provide a standardized method for assessing user satisfaction. Once fully evaluated, the questionnaire could support both developmental usability testing and postmarket evaluation, facilitating evidence-based decision-making and guiding future product improvement.

## Introduction

The field of assistive, rehabilitation, and training technologies is rapidly advancing, presenting both opportunities and challenges [1]. User satisfaction plays a critical role in the adoption, adherence, and effectiveness of these technologies, thereby directly influencing the user’s quality of life [2]. Consequently, the reliable assessment of user satisfaction and experience is essential for the successful development, provision, and implementation of such technologies. Existing assessment tools frequently rely on generic questionnaires that may not adequately capture the specific characteristics of technologies associated with clinical and training applications. This underscores the need for instruments specifically designed to evaluate user satisfaction within the domains of assistive, rehabilitation, and training technologies. The objective of this study was to describe the development and content validation of a new questionnaire for assessing user satisfaction. The intended respondents include individuals who wear, use, or train with the technology, as well as professionals who operate or provide it, such as physiotherapists, personal trainers, kinesiologists, and other health care professionals. The proposed questionnaire aims to fill an important gap in the existing literature by providing a new instrument for assessing and providing feedback on the design, usability, and effectiveness of these technologies, ultimately enhancing overall user experience and satisfaction.

Assistive, rehabilitation, and training technologies comprise tools, devices, and systems designed to maintain or enhance individuals’ functioning, participation, health, and well-being. Rehabilitative and assistive technologies facilitate the engagement of individuals with disabilities in daily activities and support caregivers by improving independence and task performance [1]. Rehabilitation technologies primarily aim to help individuals regain lost function following illness or injury, whereas assistive technologies support individuals in performing tasks that might otherwise be difficult or impossible [3]. Assistive technologies, within the scope of this study, are hardware technologies classified according to the ISO 9999:2022 classification and range from low-tech aids, such as communication boards, to advanced computer-based systems, including prosthetics, specialized keyboards, exoskeletons, and others [4,5]. Assistive technologies are often categorized by complexity and user needs as low-, medium-, or high-level systems [5]. Recent advances have expanded these categories to include wearable technologies, virtual reality, AI, and robotics, offering new opportunities to enhance independence and quality of life [1,6,7]. Some of these devices, with variations in design and intended use, are also classified as rehabilitation technologies. Examples of rehabilitation technologies include exoskeletons, robotic-assisted therapy, virtual reality rehabilitation, and electrotherapy devices, while common assistive technologies include wheelchairs, canes, hearing aids, and speech recognition software. Rehabilitation technologies can also encompass assessment and monitoring tools, such as motion capture systems and musculoskeletal modeling software, which are used in both rehabilitation and training contexts. Training technologies, on the other hand, include devices designed to maintain or improve health, well-being, and performance in individuals with or without disabilities. Examples include treadmills, stationary bicycles, rowing machines, isokinetic devices, and sport-specific systems aimed at optimizing movement patterns. Additionally, occupational training devices are used to simulate job tasks, train, and improve specific skills.

Human-centered design (HCD) is a key approach in device and system development that ensures products are usable, useful, and aligned with users’ needs by integrating principles of human factors, ergonomics, and usability [8,9]. HCD enhances product effectiveness, efficiency, user satisfaction, and accessibility while reducing potential adverse effects and promoting overall well-being [9]. According to the ISO, HCD is an iterative, cyclic process comprising 4 main phases: understanding and specifying the context of use, specifying user requirements, designing solutions, and evaluating against requirements [9]. In the development of assistive, rehabilitation, and training technologies, applying an HCD approach is essential to ensure that the final product meets the needs and preferences of end users. The ultimate goal of HCD is to create technologies that are necessary, functional, and enjoyable to use.

Usability testing is a fundamental component of the HCD process used to evaluate a product’s ease of use, efficiency, and user satisfaction through testing with representative users [10]. It ensures that systems are not only functional but also intuitive, efficient, and aligned with user needs [11]. By identifying usability issues early in development, usability testing helps reduce later design costs and supports the development of technologies that provide positive user experiences [12,13]. A well-designed usability study involves participant recruitment, task design, data collection, and systematic analysis to obtain both qualitative and quantitative insights that inform design decisions and improve user experience [10,14]. Well-structured usability testing is therefore essential for understanding user interactions, identifying relevant user experience factors and potential pain points, and detecting barriers to implementation and opportunities for improvement [15,16]. However, in practice, HCD and usability testing are often carried out without the iterative approach needed to fully follow the principles of the HCD process [17]. Insufficient involvement of end users in the design process can lead to poor user acceptance and approval, emphasizing the need for developers to effectively integrate user needs throughout the design cycle. Similarly, the absence of systematic usability evaluation and adequate consideration of users’ needs may contribute to reduced acceptance or abandonment of assistive technologies [18-20].

When planning and conducting usability testing, developers must determine the appropriate timing and frequency of testing within the design process, identify target end users, and establish methods for evaluating effectiveness, efficiency, and user satisfaction. Common approaches include structured and semistructured interviews, focus groups, surveys, scenario-based testing, expert evaluations, and controlled user observations [12]. Both qualitative and quantitative methods are essential for comprehensive usability evaluation and are often combined depending on the development stage and testing objectives. However, reliance on existing quantitative tools such as surveys and standardized questionnaires can sometimes result in narrow or incomplete insights, potentially overlooking user needs and opportunities for design improvement. Conversely, qualitative methods such as focus groups and observational studies, while rich in detail, can be time-consuming and expensive. In software usability testing, common quantitative measures include task completion metrics (with or without video or screen recording) and standardized instruments such as the System Usability Scale (SUS), Usefulness, Satisfaction, and Ease of Use (USE), or NASA Task Load Index (NASA-TLX). The literature review was conducted to identify existing instruments suitable for evaluating user experience and satisfaction with hardware and devices that interact with the human body. The identified questionnaires were systematically examined with respect to their intended purpose, target technology, and domain coverage to determine if the development of the new tool was justified. The literature review yielded similar findings to previous studies: there are a large number of custom-made instruments used for satisfaction evaluation, and the identified instruments are either too general to identify specific design issues or too specific for broader application [21-23]. To overcome these limitations, a quantitative assessment tool was designed specifically to evaluate user satisfaction with assistive, rehabilitation, and training technologies. The instrument is intended to complement qualitative methods by providing a structured and potentially cost-efficient means of assessing satisfaction across individual items, broader domains, and overall device use. Following further validation steps, the questionnaire may be suitable not only for researchers and developers during the design process but also for routine commercial or clinical use of the device after development, as well as for postmarket evaluation. In these contexts, it could support the ongoing evaluation of user experience and satisfaction throughout the product life cycle, thereby informing continuous improvement and future design iterations. Additionally, it could assist researchers and clinicians in comparing user satisfaction across devices, settings, and user populations, thereby informing evidence-based decision-making in device selection and provision.

During the development process of the User Satisfaction Questionnaire for Assistive, Rehabilitation, and Training Technologies (U-ART-Q), it was considered essential to account for the distinct characteristics, intended purposes, and needs of end users of assistive, rehabilitation, and training technologies.

As emphasized in previous studies, comprehensive coverage, item clarity, and precise definition of measured constructs are crucial for creating valid and usable measurement instruments [2]. While detailed psychological definitions of satisfaction are beyond the scope of this study, user satisfaction in this context is conceptualized as a component of user experience, in line with the ISO definition: “Users’ perceptions and responses that result from the use and/or anticipated use of a system, product or service.” Satisfaction itself is defined as “the extent to which the user’s physical, cognitive and emotional responses that result from the use of a system, product or service meet the user’s needs and expectations” [9].

## Methods

### Overview

**Figure 1.**
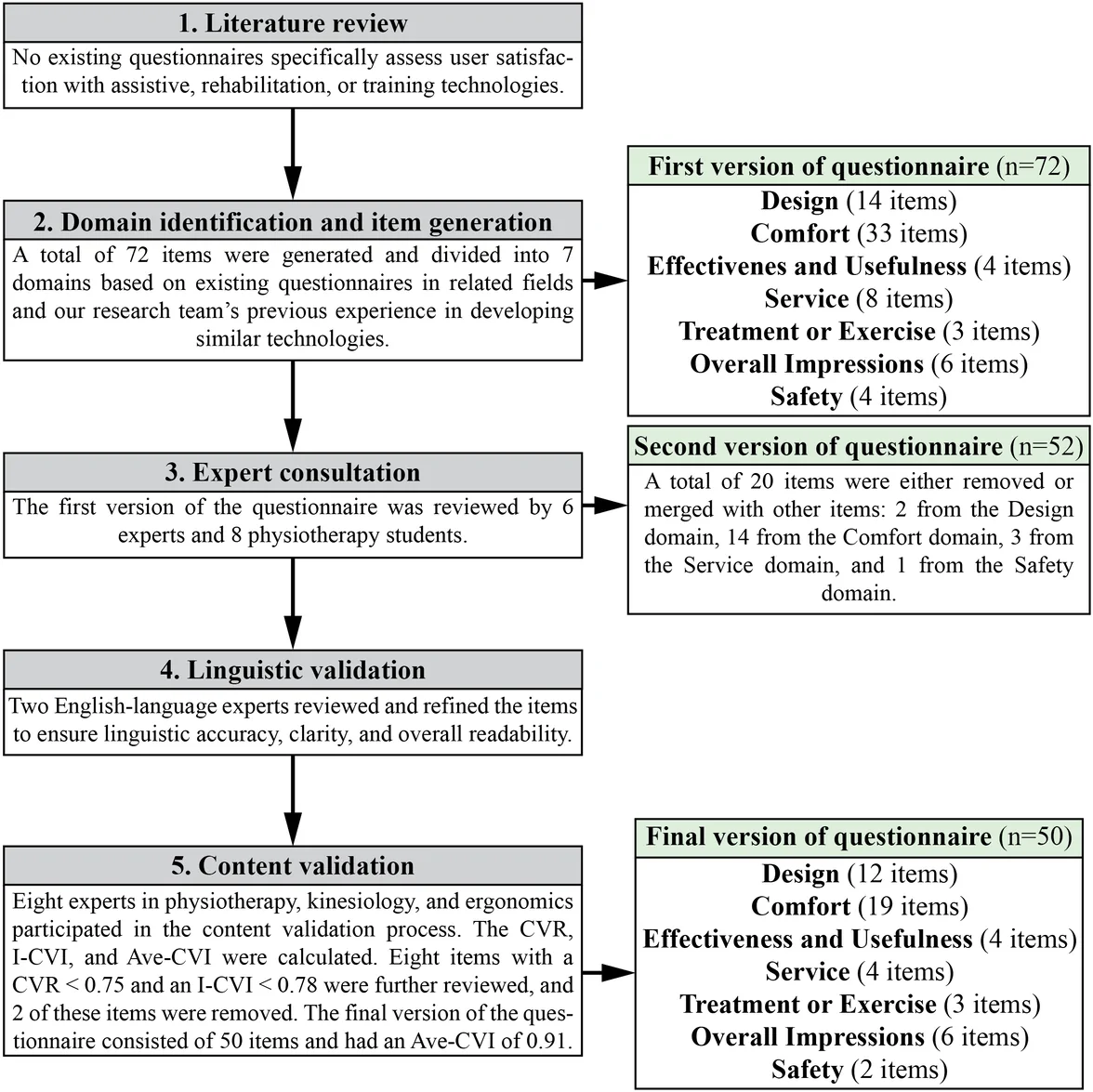
Development process of the User Satisfaction Questionnaire for Assistive, Rehabilitation, and Training Technologies (U-ART-Q). Ave-CVI: average content validity index; CVR: content validity ratio; I-CVI: item-level content validity index.

The development process of the U-ART-Q involved several distinct phases, including (1) literature review, (2) domain definition and item generation, (3) expert consultation, (4) linguistic validation, and (5) content validation. The detailed development process of this questionnaire is presented in Figure 1.

The first step in developing the U-ART-Q was to conduct a literature review to identify existing questionnaires assessing user satisfaction with assistive, rehabilitation, and training technologies. The search was conducted in the PubMed/MEDLINE, Scopus, and Web of Science databases on September 10, 2025. Database-specific search strategies were developed using combinations of terms related to satisfaction, user experience, questionnaires, development, rehabilitation, assistive technology, robotic rehabilitation, exoskeletons, prostheses, orthoses, and training technologies. The complete search strategies for each database are presented in Multimedia Appendix 1.

All retrieved records were exported to a Mendeley Reference Manager (Elsevier), and duplicate records were removed. Titles and abstracts were independently screened according to the predefined eligibility criteria. Potentially relevant studies underwent full-text review to determine final eligibility. Reasons for exclusion during the full-text assessment were documented in accordance with PRISMA (Preferred Reporting Items for Systematic Reviews and Meta‑Analyses) recommendations [24].

Studies were considered eligible if they reported the development, validation, adaptation, or application of questionnaires intended to evaluate user satisfaction with relevant technologies. Furthermore, studies were excluded if they focused on technologies outside the scope of rehabilitation, such as industrial exoskeletons, surgical robotics, dental technologies, and other unrelated medical devices. As our search was based only on hardware solutions, publications evaluating software or mobile apps without other hardware components were also excluded. Similarly, user interfaces without accompanying hardware components and virtual reality systems were excluded. Additionally, studies that evaluated other constructs without measuring satisfaction (eg, quality of life, functional performance, technology acceptance without satisfaction outcomes, or clinical effectiveness alone) were excluded. Conference abstracts without full text available, editorials, letters, study protocols, dissertations, non-English publications, and studies with insufficient methodological information were also excluded.

Following full-text screening, studies were excluded for the following reasons: published study not in English language (n*=*9); technology outside the scope of this review (n=16); no satisfaction-related outcome measures reported (n=64); and study that does not use a questionnaire (n=3).

The complete study selection process is illustrated in the PRISMA flow diagram. All included studies are presented in Multimedia Appendix 2 (Microsoft Excel worksheet titled Studies for Extraction).

After searching the literature, we noted the existence of 117 questionnaires used to measure user experience, usability, and satisfaction with various technologies and systems. The most frequently used questionnaires were general questionnaires, such as the Quebec User Evaluation of Satisfaction With Assistive Technology (QUEST 2.0), USE, and SUS, as well as questionnaires specific to certain technologies, such as the Client Satisfaction With Device Module of the Orthotics and Prosthetics Users’ Survey (CSD-OPUS), the Questionnaire for Usability Evaluation of orthopaedic shoes (QUE), the Trinity Amputation and Prosthesis Experience Scale (TAPES), the prosthesis-satisfaction subscale, and others [23,25-31]. The list of all identified questionnaires used in the evaluation of satisfaction with technologies is available in Multimedia Appendix 2 (Excel worksheet titled Identified Questionnaires).

Although a considerable number of questionnaires were identified (Multimedia Appendix 2), several limitations were noted. The identified questionnaires were either designed for a specific type of technology or failed to cover all important aspects necessary to provide detailed information on user satisfaction with assistive, rehabilitation, and training technologies. Forty-six out of 117 identified questionnaires were completely custom questionnaires, intended for the specific device or population in the study, limiting their broader applicability. Eighty-nine out of 117 questionnaires were developed specifically for the needs of the study, including custom questionnaires, modified instruments for usability testing of specific devices, newly developed questionnaires, and translations of existing questionnaires. Only 23 out of 117 questionnaires were reported in more than a single publication. Among the identified questionnaires, 58 primarily evaluated usability, user experience, technology acceptance, or overall system performance rather than user satisfaction, with satisfaction represented by only a small number of items instead of a comprehensive multidimensional construct. Additionally, none of the questionnaires appeared to cover all identified satisfaction domains. Item-level analysis demonstrated that domains with similar labels (eg, design) frequently assessed different underlying constructs and therefore did not capture equivalent aspects of user satisfaction. The identified questionnaires were also developed and used for different technology categories: 84 for assistive, 23 for rehabilitation, 9 for assistive and rehabilitation, 1 for nontechnology-specific questionnaire, and none for training technologies (Multimedia Appendix 2). Although several questionnaires have subsequently been applied beyond their original context, they were not specifically developed for these broader applications. In addition, widely used generic instruments, such as the SUS, were designed as technology-independent usability measures rather than for evaluating hardware-based technologies that directly interact with the human body and therefore do not capture several device-specific aspects identified as important in this review. Overall, while the identified questionnaires provide valuable information on specific aspects of user evaluation, no single instrument was found to simultaneously (1) assess user satisfaction as its primary outcome; (2) comprehensively cover all identified satisfaction domains at the item level; and (3) be specifically developed for application across assistive, rehabilitation, and training technologies.

Therefore, we aimed to develop a questionnaire to comprehensively assess user satisfaction across different types of assistive, rehabilitation, and training technologies. The questionnaire was specifically designed to capture the perspectives of direct end users, including both individuals using or training with the technology and professionals such as physiotherapists, kinesiologists, personal trainers, and other health care providers involved in its operation and delivery.

The second phase of the development process focused on identifying key domains and generating items to evaluate user experience and satisfaction with assistive, rehabilitation, and training technologies. The initial version of the questionnaire consisted of 72 items divided into 7 thematic domains. Item generation was guided by the preceding literature review and further informed by the research team’s prior experience in developing and evaluating similar technologies. The list of all identified items and related domains during the literature review is available in Multimedia Appendix 2 (Excel worksheet titled Items and Domains). Items were generated with the intention that each item should apply to at least one type of existing assistive, rehabilitation, or training technology. However, given that such technologies can differ significantly in their fundamental characteristics, not all items are relevant for every device. To address this, a set of initial screening questions was developed to be completed by the researcher or developer prior to administering the questionnaire. The responses to these screening questions are used to generate a tailored set of questionnaire items, selected according to the characteristics of the technology and the role of the end user (eg, patient, client, therapist, or trainer). Furthermore, the researcher or developer may manually adjust the recommended item set by adding or removing questions to ensure optimal relevance for the specific technology.

After the domains were identified and the items generated, a round of expert revision was conducted. Six experts with extensive experience in the design and evaluation of assistive, rehabilitation, and training technologies and 8 master’s students in physiotherapy were recruited to evaluate each item independently as either “relevant” or “not relevant.” Additional information about the experts included in the expert consultation is presented in Table 1. For any item marked as not relevant, the recruited experts were asked to provide a brief explanation for their decision. Based on the results obtained, the questionnaire was revised, and items marked as not relevant were either removed or modified. Following the revision process, the questionnaire was evaluated by 2 English-language experts to ensure linguistic accuracy, clarity, and overall readability.

**Table 1.** Additional information about the expert panel included in the expert consultation.

ID	Years of work experience	Employer	Field of research/work
E1	23	National research institute	Collaborative robotics
E2	16	National research institute	Collaborative robotics
E3	9	National research institute	Humanoid and cognitive robotics
E4	20	Clinical institution	Rehabilitation medicine
E5	27	Higher education institution	Engineering design
E6	12	Industry/private sector	Sports products development

The final part of the development process was the evaluation of content validity. Content validity relates to the degree to which a sample of items constitutes an adequate operational definition of a concept [32]. To assess item content validity, another 8 experts in physiotherapy, kinesiology, and ergonomics were recruited. These experts were independent of the 6 experts involved in the previous stage of questionnaire development and had at least 5 years of experience in the development or use of assistive, rehabilitation, or training technologies. The participating experts were informed of the purpose of the questionnaire and its intended use to evaluate user experience and satisfaction with the assistive, rehabilitation, and training technologies. Each expert independently evaluated all items of the questionnaire on a 4-point Likert-type scale (1=“the item is not relevant to the measured domain,” 2=“the item is somewhat relevant to the measured domain,” 3=“the item is quite relevant to the measured domain,” and 4=“the item is highly relevant to the measured domain”). The content validity ratio (CVR) was calculated for each item alongside the content validity index for each item (I-CVI) using equations 1 and 2, where *n*_4_ represents the number of experts grading an item as highly relevant (grade 4), *n*_3,4_ represents the number of experts grading an item as quite or highly relevant (grade 3 or 4), and *N* represents the total number of experts. Additionally, the average content validity index (Ave-CVI) was calculated as the average of the I-CVIs, representing the content validity of the overall instrument [33].


(1) $$CVR=\frac{\left({\text{n}}_{\text{e}}-\frac{\text{N}}{2}\right)}{\left(\frac{\text{N}}{2}\right)}$$

(2) $$I\text{-}CVI=\frac{{\text{n}}_{3,4}}{\text{N}}$$

Items with a CVR below 0.75 and an I-CVI below 0.78 were reviewed and either revised or removed in accordance with the content validity criteria described by Lawshe [34] and Lynn [35].

### Ethical Considerations

All participants received oral and written information about the study, were informed that participation was voluntary, and provided informed consent prior to participation. Participant confidentiality was ensured throughout the study. The study was approved by the University of Primorska Commission for Ethics in Research Involving Human Subjects (approval number: 4264-19-6/23) and was conducted in accordance with the principles of the Declaration of Helsinki.

## Results

**Figure 2.**
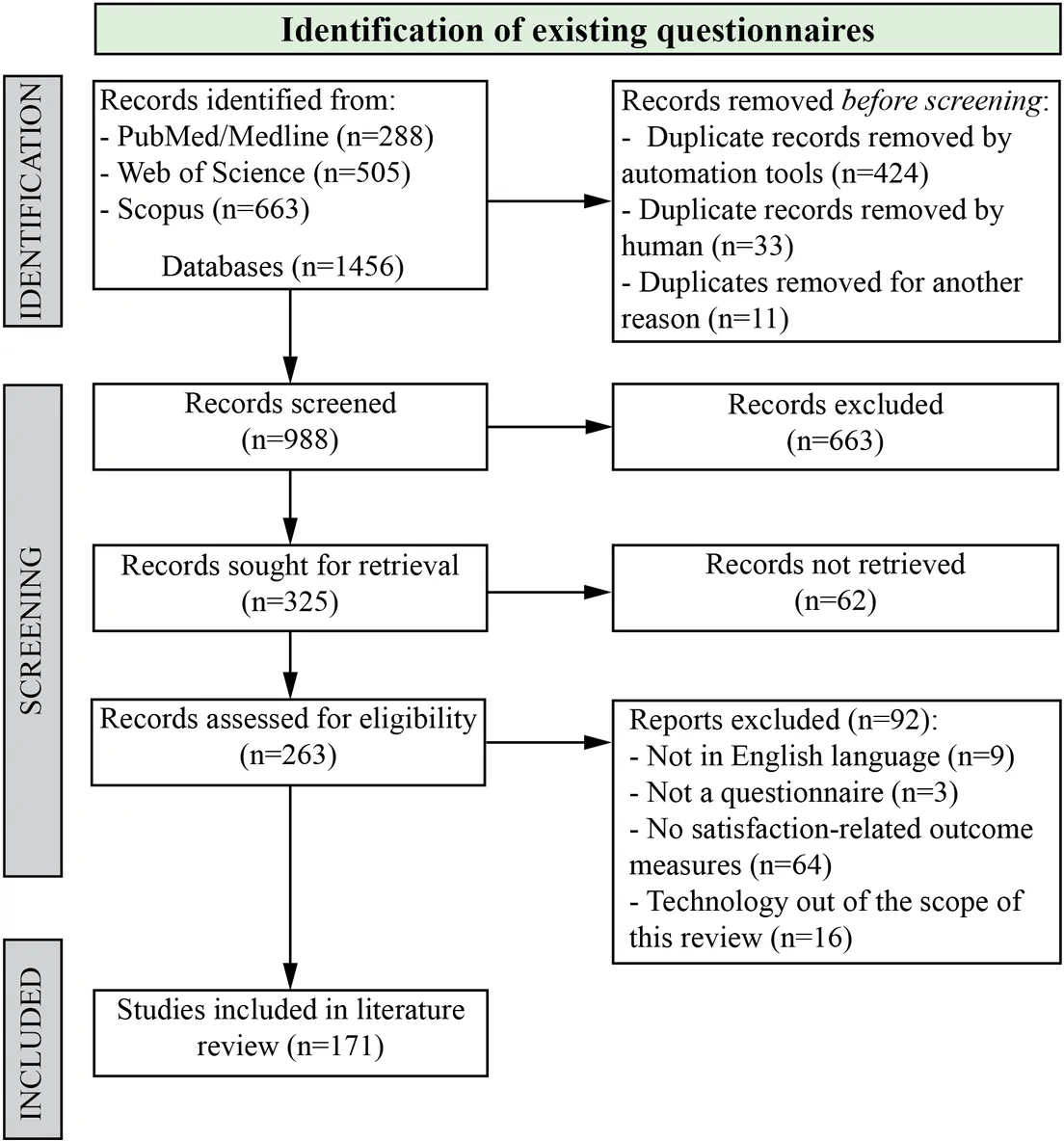
PRISMA (Preferred Reporting Items for Systematic Reviews and Meta‑Analyses) flow diagram for the literature review.

Following an initial literature review (the complete study selection process is illustrated in the PRISMA flow diagram Figure 2) and drawing on the research team’s experience with similar technological developments, the first version of the questionnaire was developed, comprising 72 items divided into 7 domains. The identified domains were as follows: design (D), comfort (C), effectiveness and usefulness (EU), service (SE), treatment or exercise (TE), overall impressions (OI), and safety (SA).

Once the first version of the U-ART-Q was prepared, it was reviewed by 6 experts with extensive experience in the design and evaluation of assistive, rehabilitation, and training technologies and 8 master’s students in physiotherapy. Based on their feedback, 2 items from design, 14 from comfort, 3 from service, and 1 from safety domain were either removed or merged with other items to reduce redundancy and improve clarity.

The refined version of the questionnaire was further reviewed and revised by 2 English-language experts to improve accuracy, clarity, and overall readability. Content validity was then assessed based on an evaluation by 8 experts in physiotherapy, kinesiology, and ergonomics. Of the 52 items, 44 met the predefined CVR values >0.75 and I-CVI values >0.78 and were therefore considered suitable for inclusion in the final questionnaire. The remaining 8 items, with CVR values <0.75 and I-CVI values <0.78, were subjected to additional review and are presented in Table 2.

**Table 2.** Items with CVR^a^ or I-CVI^b^ values below predefined thresholds and corresponding actions taken.

Item	CVR	I-CVI	Action
D9^c^: I am satisfied with the battery life of the assistive/rehabilitation/training technology.	0.5	0.75	Retained
D12^d^: I am satisfied with the design and appearance of the assistive/rehabilitation/training technology.	0.5	0.75	Retained
C6^e^: I am satisfied with the comfort of the contact point/points in the upper arm area.	0.5	0.75	Retained
C10^f^: I am satisfied with the comfort of the contact point/points in the wrist area.	0.5	0.75	Retained
SE2^g^: I am satisfied with the time needed to understand how the assistive/rehabilitation/training technology is supposed to operate/function.	0.5	0.75	Retained
SE4^h^: I am satisfied with the performance of the assistive/rehabilitation/training technology in the specified weather conditions.	0.25	0.625	Removed
OI3^i^: The assistive/rehabilitation/training technology is pleasant to use.	0.5	0.75	Retained
SA2^j^: I have experienced no anxiety when using the assistive/rehabilitation/training technology.	0.25	0.625	Removed

a CVR: content validity ratio.

b I-CVI: item-level content validity index.

c D9: item number 9 from the design domain.

d D12: item number 12 from the design domain.

e C6: item number 6 from the comfort domain.

f C10: item number 10 from the Comfort domain.

g SE2: item number 2 from the service domain.

h SE4: item number 4 from the service domain.

i OI3: item number 3 from the overall impressions domain.

j SA2: item number 2 from the safety domain.

Following this review, 6 of these items were retained because they represented important aspects of the construct that were not adequately covered by other items. For example, although item D9, which refers to battery life, had CVR and I-CVI values below the predefined thresholds, it was retained because battery life was considered a highly relevant aspect of user satisfaction with technologies that operate on battery power [36]. Item D12, related to satisfaction with the appearance of the device, was retained, as satisfaction with appearance was identified in the literature as an important factor influencing user satisfaction, product abandonment, adoption, and long-term use [37-39]. Additionally, items regarding the appearance of the device were identified in 31 questionnaires during the literature review; therefore, this item was retained as part of the design domain. Items C6 and C10 were retained because all other items related to comfort at the contact points were rated as relevant. Because the upper arm or wrist may be the only contact points for some technologies, eliminating these items would limit the applicability of the questionnaire across different types of technologies. Regarding the retention of item OI3, the rationale was that user satisfaction encompasses the user’s emotional response arising from interacting with a product or system. Therefore, perceived pleasantness represents an important affective dimension of satisfaction and was considered essential to retain in the questionnaire. The current definition of satisfaction in ISO 9241-11:2018 [40] explicitly encompasses users’ physical, cognitive, and emotional responses, while ISO/IEC 25019:2023 [41] identifies user satisfaction as an emotional and hedonic outcome in which a pleasant, positive subjective experience is essential. In contrast, item SA2 was removed, even though anxiety was included as an item in some satisfaction questionnaires. The decision to remove this item was based on the fact that anxiety is a distinct psychological construct for which dedicated instruments exist, whereas U-ART-Q is intended to assess satisfaction. There are many other standardized tools, such as the Abbreviated Technology Anxiety Scale (ATAS), Computer Anxiety Rating Scale (CARS), Technology Assisted Rehabilitation Patient Perception Questionnaire (TARPP-Q), and the General Anxiety Disorder 7-item screening tool, developed for the comprehensive assessment of technophobia and anxiety symptoms [42-45]. The item assessing satisfaction with the performance of the training technology under the specified weather conditions (SE4) was identified in only a small number of questionnaires, and insufficient evidence was found in the literature to justify its retention. Therefore, the item was removed. After removing these 2 items (SA2 and SE4), the final Ave-CVI, calculated across the remaining 50 items, was 0.915, which is above the acceptable standard reported in the literature. A complete list of all 10 initial screening questions and the final 50 items of the U-ART-Q is provided in Multimedia Appendix 3.

**Figure 3.**
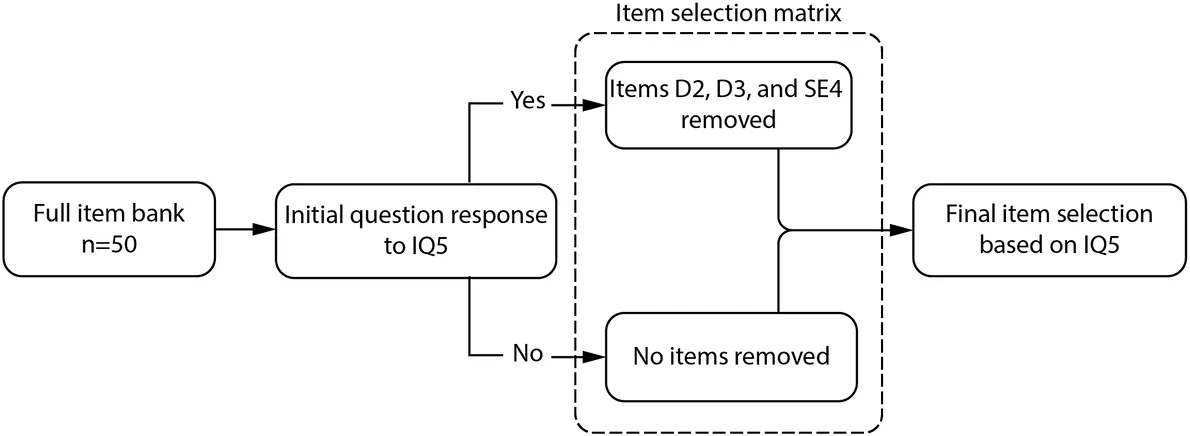
Example of the item selection process for initial screening question number 5 (IQ5).

An additional set of 10 initial screening questions was created to determine the applicability of specific items based on the type of technology being evaluated. These initial questions are completed by the researcher who intends to use the questionnaire and are designed to identify which items are relevant, depending on the technology and the end user. Final questionnaire items are selected using the question selection matrix that is available in Multimedia Appendix 4. Each column of the matrix represents one possible response to an initial screening question, and each row represents 1 final questionnaire item. For each selected response, the corresponding matrix column is applied as a filter: items coded as 1 are retained, and items coded as 0 are excluded from the final questionnaire. Figure 3 illustrates the selection process for one of the screening questions. When the response to IQ5 is “Yes,” the final questions Q4, Q5, Q39, and Q40 are removed, while all remaining items are retained.

Items in the developed questionnaire are formulated in such a way that they can be graded on a 5-point Likert scale. The items can be graded as 1=“strongly disagree,” 2=“disagree,” 3=“unsure,” 4=“agree,” and 5=“strongly agree.”

To support the practical implementation of the item-selection process, a macro-enabled Excel tool (.xlsm) was developed and is provided in Multimedia Appendix 5. The purpose of this tool is to automatically generate the final set of questionnaire items based on responses to the initial screening questions. The tool is organized into separate worksheets. The worksheet named “Instructions” provides a step-by-step guide on how to use the Excel file to generate the final item selection. The “Initial_Questions” worksheet allows the user to select the responses to the initial questions from a drop-down list, thereby minimizing the possibility of incorrect entries. After all responses to the initial questions have been selected, the user can click the “Create_Final_Question Selection” button. The final questionnaire is then generated in the “Final_Item_Selection” worksheet. Following review, the generated U-ART-Q questionnaire can be exported as a PDF, printed, and used in practical applications.

## Discussion

### Principal Findings

HCD is a vital component of the product development process, ensuring that technologies are designed around the actual needs, abilities, and expectations of end users. In assistive, rehabilitation, and training technologies, HCD promotes usability, accessibility, and satisfaction by actively involving end users throughout the design, testing, and implementation phases. A key component of HCD is usability testing, which provides critical insight into how users interact with a product and identifies barriers to effective use. Failing to include usability testing at different stages of development can result in technically advanced products that do not meet user expectations, leading to poor acceptance, limited adoption, and ultimately unsuccessful implementation.

Although several relevant questionnaires were identified in the literature, none was considered fully suited to the specific purpose and scope of this study. Therefore, this study introduces the newly developed U-ART-Q and evaluates its content validity. It serves as a complementary tool for evaluating user satisfaction with assistive, rehabilitation, and training technologies. The items and domains for the initial version of the questionnaire were identified through a comprehensive literature review. The preliminary set of items and domains underwent expert validation, which resulted in the removal or merging of 20 items to reduce redundancy and improve clarity. To further enhance linguistic accuracy, clarity, and overall readability, the revised questionnaire was reviewed and refined by 2 English-language experts. Finally, 8 additional experts with backgrounds in physiotherapy, kinesiology, and ergonomics participated in the content validation process. Out of 52 items, 44 met the predefined CVR and I-CVI thresholds and therefore met the predefined criteria for content validity. The remaining 8 items with CVR and I-CVI values below the recommended thresholds were reviewed further, resulting in the removal of 2 items from the item bank. Finally, the remaining 50-item questionnaire achieved a high Ave-CVI of 0.915, demonstrating strong evidence of content validity based on expert evaluation.

The proposed U-ART-Q represents a newly developed questionnaire with evidence of content validity for assessing user satisfaction within the domains of assistive, rehabilitation, and training technologies. To ensure its relevance across a broad range of technologies, the researcher or developer administering the questionnaire first completes a set of initial screening questions that guide item selection. This approach allows the instrument to be tailored to the specific characteristics of the technology being evaluated, making it both concise and practical for use across diverse assistive, rehabilitation, and training technologies.

The proposed questionnaire offers several important contributions to both research and practice. For researchers and clinicians, it is intended to serve as a structured and efficient evaluation tool for existing technologies, allowing user satisfaction to be compared across different devices, applications, and user groups. Its use can contribute to a more comprehensive understanding of user experience, leading to more informed decisions regarding device selection, clinical implementation, and future product improvement. For developers, the questionnaire provides a structured and standardized tool for collecting user feedback throughout the design process. It helps identify usability issues early and supports evidence-based design decisions. By systematically capturing satisfaction across multiple domains, the instrument can help optimize the development process by reducing redesign costs and improving the functionality of technologies under development. As user satisfaction and experience are closely linked to successful technology implementation, the results may also help clinical managers and other stakeholders compare available market options and support the decision-making, selection, and purchasing of technologies.

Future work will focus on further validation and practical application of the questionnaire. Pilot testing will be conducted to evaluate reliability, internal consistency, and test-retest reliability, and validated translations of the questionnaire into Serbian and Slovenian will be completed. Although a macro-enabled Excel tool (.xlsm) has been developed to support automated item selection, which is provided in Multimedia Appendix 5, the development of a mobile app or a web-based version of the questionnaire is planned to facilitate simple, time-efficient, and cost-effective administration in clinical and research settings.

### Limitations

The development of the U-ART-Q has several limitations. First, the existing literature did not provide a comprehensive conceptual framework that specifically describes the multidimensional structure of user satisfaction with assistive, rehabilitation, and training technologies. Consequently, the domains and items of the U-ART-Q were informed by findings from the literature, existing questionnaires, expert consultations, and the research team’s experience in developing similar technologies. Although the experts involved had substantial experience in relevant fields, expert-based evaluation is inherently subject to some degree of subjectivity. Different expert panels could have reached different conclusions regarding the relevance, wording, or importance of individual items.

Additionally, it is important to point out that the U-ART-Q is a newly developed questionnaire with initial evidence of content validity. No actual end users were included in the development or content-validation process, which represents an important limitation, particularly because the questionnaire is intended to assess user satisfaction. As such, the U-ART-Q should undergo further evaluation before it can be considered a fully validated and standardized instrument for assessing user satisfaction with assistive, rehabilitation, and training technologies. In line with recommended questionnaire-development procedures, important next steps include face validity assessment with intended users, pilot testing, evaluation of construct validity (through exploratory and confirmatory factor analysis), reliability testing, and establishment of a clear scoring system and interpretation of the results [46].

### Conclusions

In conclusion, the U-ART-Q is a newly developed questionnaire with evidence of content validity. Its main objective is to support researchers and developers applying HCD principles in the evaluation and development of assistive, rehabilitation, and training technologies. The questionnaire is intended to complement existing methods of assessing user experience, including interviews, focus groups, and observational approaches. By integrating this quantitative measure with qualitative methods, the U-ART-Q can contribute to more informed, iterative, and end user–driven design processes. Future work will focus on further psychometric evaluation needed to determine whether the U-ART-Q can be established as a validated and standardized questionnaire for assessing user satisfaction with assistive, rehabilitation, and training technologies.

## Supplementary material

10.2196/88182 Multimedia Appendix 1 Literature review search strategies for each database.

10.2196/88182 Multimedia Appendix 2 Excel file containing additional information about identified studies and questionnaires.

10.2196/88182 Multimedia Appendix 3 Complete item bank containing all items developed for the Questionnaire for Assistive, Rehabilitation, and Training Technologies (U-ART-Q).

10.2196/88182 Multimedia Appendix 4 Question selection matrix.

10.2196/88182 Multimedia Appendix 5 Excel-based questionnaire generator (.xlsm) that uses initial responses to produce a tailored list of items, which can be viewed and exported to PDF.

## Abbreviations

ATAS: Abbreviated Technology Anxiety Scale
CARS: Computer Anxiety Rating Scale
CSD-OPUS: Client Satisfaction With Device Module of the Orthotics and Prosthetics Users’ Survey
CVR: content validity ratio
HCD: human-centered design
I-CVI: item-level content validity index
NASA-TLX: NASA Task Load Index
PRISMA: Preferred Reporting Items for Systematic Reviews and Meta‑Analyses
QUE: Questionnaire for Usability Evaluation of shoes
QUEST 2.0: Quebec User Evaluation of Satisfaction With Assistive Technology
SUS: System Usability Scale
TAPES: Trinity Amputation and Prosthesis Experience Scale
TARPP-Q: Technology Assisted Rehabilitation Patient Perception Questionnaire
U-ART-Q: User Satisfaction Questionnaire for Assistive, Rehabilitation, and Training Technologies
USE: Usefulness, Satisfaction, and Ease of Use
