# Supplementary material for: User Satisfaction With Assistive, Rehabilitation, and Training Technologies: Questionnaire Development and Content Validation Study
Source: JMIR Rehabil Assist Technol. 2026 Sep 25;13:e88182. doi: 10.2196/88182 (PMC13614623; doi:10.2196/88182)
Supplement: Supplementary file 1 [file rehab-v13-e88182-s004.pdf]

Question Selection Matrix

Question ID	IQ2		IQ3			IQ4		IQ5		IQ6		IQ7		IQ8		IQ9		IQ10	
	HP / Tr	Pa / Ex	AT	RT	ET	Y	N	Y	N	Y	N	Y	N	Y	N	Y	N	Y	N
**D1**	1	1	1	1	1	1	1	1	1	1	1	1	1	1	1	1	1	1	1
**D2**	1	1	1	1	1	1	1	0	1	1	1	1	1	1	1	1	1	1	1
**D3**	1	1	1	1	1	0	1	0	1	1	1	1	1	1	1	1	1	1	1
**D4**	1	0	1	1	1	1	1	1	1	1	1	1	1	1	0	1	1	1	1
**D5**	0	1	1	1	1	1	1	1	1	1	1	1	1	1	1	1	1	1	1
**D6**	1	1	1	1	1	1	1	1	1	1	1	1	1	1	1	1	1	1	1
**D7**	1	1	1	1	1	1	1	1	1	1	1	1	0	1	1	1	1	1	1
**D8**	1	1	1	1	1	1	1	1	1	1	1	1	1	1	1	1	1	1	1
**D9**	1	1	1	1	1	1	1	1	1	1	1	1	1	1	1	1	1	1	0
**D10**	0	1	1	1	1	1	1	1	1	1	1	1	1	1	1	1	1	1	1
**D11**	1	1	1	1	1	1	1	1	1	1	1	1	1	1	1	1	1	1	1
**D12**	1	1	1	1	1	1	1	1	1	1	1	1	1	1	1	1	1	1	1
**C1**	0	1	1	1	1	1	1	1	1	1	0	1	1	1	1	1	1	1	1
**C2**	0	1	1	1	1	1	1	1	1	1	0	1	1	1	1	1	1	1	1
**C3**	0	1	1	1	1	1	1	1	1	1	0	1	1	1	1	1	1	1	1
**C4**	0	1	1	1	1	1	1	1	1	1	0	1	1	1	1	1	1	1	1
**C5**	0	1	1	1	1	1	1	1	1	1	0	1	1	1	1	1	1	1	1
**C6**	0	1	1	1	1	1	1	1	1	1	0	1	1	1	1	1	1	1	1
**C7**	0	1	1	1	1	1	1	1	1	1	0	1	1	1	1	1	1	1	1
**C8**	0	1	1	1	1	1	1	1	1	1	0	1	1	1	1	1	1	1	1
**C9**	0	1	1	1	1	1	1	1	1	1	0	1	1	1	1	1	1	1	1
**C10**	0	1	1	1	1	1	1	1	1	1	0	1	1	1	1	1	1	1	1
**C11**	0	1	1	1	1	1	1	1	1	1	0	1	1	1	1	1	1	1	1
**C12**	0	1	1	1	1	1	1	1	1	1	0	1	1	1	1	1	1	1	1
**C13**	0	1	1	1	1	1	1	1	1	1	0	1	1	1	1	1	1	1	1
**C14**	0	1	1	1	1	1	1	1	1	1	0	1	1	1	1	1	1	1	1
**C15**	0	1	1	1	1	1	1	1	1	1	0	1	1	1	1	1	1	1	1
**C16**	0	1	1	1	1	1	1	1	1	1	0	1	1	1	1	1	1	1	1
**C17**	0	1	1	1	1	1	1	1	1	1	0	1	1	1	1	1	1	1	1
**C18**	0	1	1	1	1	1	1	1	1	1	0	1	1	1	1	1	1	1	1

Only Include Body parts selected in IQ6 (C3–C18)

Question Selection Matrix

**C19**	0	1	1	1	1	1	1	1	1	1	0	1	1	1	1	1	1	1	1
**EU1**	1	1	1	1	1	1	1	1	1	1	1	1	1	1	1	1	1	1	1
**EU2**	1	1	1	1	1	1	1	1	1	1	1	1	1	1	1	1	1	1	1
**EU3**	1	1	1	1	1	1	1	1	1	1	1	1	1	1	1	1	1	1	1
**EU4**	0	1	1	0	0	1	1	1	1	1	1	1	1	1	1	1	1	1	1
**SE1**	1	1	1	1	1	1	1	1	1	1	1	1	1	1	1	1	1	1	1
**SE2**	1	1	1	1	1	1	1	1	1	1	1	1	1	1	1	1	1	1	1
**SE3**	1	1	1	1	1	1	1	1	1	1	1	1	1	1	1	1	1	1	1
**SE4**	1	1	1	1	1	1	1	0	1	1	1	1	1	1	1	1	1	1	1
**TE1**	1	1	0	1	1	1	1	1	1	1	1	1	1	1	1	1	1	1	1
**TE2**	1	1	0	1	1	1	1	1	1	1	1	1	1	1	1	1	1	1	1
**TE3**	1	1	0	1	1	1	1	1	1	1	1	1	1	1	1	1	1	1	1
**OI1**	1	1	1	1	1	1	1	1	1	1	1	1	1	1	1	1	1	1	1
**OI2**	1	1	1	1	1	1	1	1	1	1	1	1	1	1	1	1	1	1	1
**OI3**	1	1	1	1	1	1	1	1	1	1	1	1	1	1	1	1	1	1	1
**OI4**	1	1	1	1	1	1	1	1	1	1	1	1	1	1	1	1	1	1	1
**OI5**	1	1	1	1	1	1	1	1	1	1	1	1	1	1	1	1	1	1	1
**OI6**	1	1	1	1	1	1	1	1	1	1	1	1	1	1	1	1	1	1	1
**SA1**	0	1	1	1	1	1	1	1	1	1	1	1	1	1	1	1	1	1	1
**SA2**	0	1	1	1	1	1	1	1	1	1	1	1	1	1	1	1	1	1	1
**SA3**	0	1	1	1	1	1	1	1	1	1	1	1	1	1	1	1	1	1	1
