# Supplementary material for: User Satisfaction With Assistive, Rehabilitation, and Training Technologies: Questionnaire Development and Content Validation Study
Source: JMIR Rehabil Assist Technol. 2026 Sep 25;13:e88182. doi: 10.2196/88182 (PMC13614623; doi:10.2196/88182)
Supplement: Supplementary file 2 [file rehab-v13-e88182-s001.docx]

The following document contains three tables: 1. List of eligibility criteria for the review used to decide whether a study was eligible for inclusion in the review; 2. The list of Keywords and their synonyms used for the development of the search strings to conduct the literature review on questionnaires assessing satisfaction with Assistive, Rehabilitation, and Training Technologies; 3. Developed Search Strings to conduct the literature review, adapted to three Databases used for the literature review.

| Inclusion Criteria | Exclusion Criteria |
| --- | --- |
| Studies published until 10^th^ of September 2025 | Study does not use a questionnaire or survey for satisfaction measurement (e.g. focus groups and other qualitative measures) |
| Studies published in English language | Used instrument does not measure satisfaction (e.g. measuring other usability or user experience aspects, but not satisfaction) |
| Study reports the Development, validation, translation, assessment or Use of the instrument intended for measurement of satisfaction with Assistive, Rehabilitation or Training Technology | Technology assessed in the study is out of the scope of this review (e.g. service robots, social robots, surgery and dentistry medical devices, implants, softwares, interfaces or mobile apps without any other hardware parts, virtual reality systems, army and defense technologies, industry and manufacturing devices, smart homes) |
| Original papers | Telerehabilitation programmes, Video manuals, Learning programmes |

Table 1 List of Inclusion and Exclusion criteria used for determining the eligibility of the studies for inclusion in the review

| List of Keywords | Synonyms |
| --- | --- |
| Satisfaction | Satisfaction, User Satisfaction, Patient Satisfaction, Usability, Acceptability, User Experience |
| Questionnaire | Questionnaire, Scale, Instrument, Survey, Inventory, Checklist |
| Development | Validation, Validity, Reliability, Psychometric, Development, Content Validity, Construct Validity, Factor Analysis, Internal Consistency |
| Assistive, Rehabilitation, Training Technology | Assistive Technology, Rehabilitation Technology, Training Technology, Assistive Device, Rehabilitation Device, Exoskeleton, Rehabilitation Robot, Assistive Robot, Orthosis, Wheelchair, Prosthetic Limb, Prosthetic Device |

Table 2 List of the Keywords and related synonyms used to develop search strings for conducting the literature review

| Data Base | Search String |
| --- | --- |
| PubMed/Medline | (  satisfaction[tiab]  OR "user satisfaction"[tiab]  OR "patient satisfaction"[tiab]  OR usability[tiab]  OR acceptability[tiab]  OR "user experience"[tiab]  )  AND  (  questionnaire*[tiab]  OR scale*[tiab]  OR instrument*[tiab]  OR survey*[tiab]  OR inventory[tiab]  OR checklist*[tiab]  )  AND  (  validation[tiab]  OR validity[tiab]  OR reliability[tiab]  OR psychometric*[tiab]  OR development[tiab]  OR "content validity"[tiab]  OR "construct validity"[tiab]  OR "factor analysis"[tiab]  OR "internal consistency"[tiab]  )  AND  (  "assistive technolog*"[tiab]  OR "rehabilitation technolog*"[tiab]  OR "training technolog*"[tiab]  OR "assistive device*"[tiab]  OR "rehabilitation device*"[tiab]  OR exoskeleton*[tiab]  OR "rehabilitation robot*"[tiab]  OR "assistive robot*"[tiab]  OR orthos*[tiab]  OR wheelchair*[tiab]  OR "prosthetic limb*"[tiab]  OR "prosthetic device*"[tiab]  )  NOT  (  dental[tiab]  OR dentistry[tiab]  OR orthodont*[tiab]  OR oral[tiab]  OR maxillofacial[tiab]  OR surgical[tiab]  OR surgery[tiab]  OR industrial[tiab]  OR manufacturing[tiab]  OR army[tiab]  ) |
| Web Of Science | TS=(  satisfaction  OR "user satisfaction"  OR "patient satisfaction"  OR usability  OR acceptability  OR "user experience"  )  AND  TS=(  questionnaire*  OR scale*  OR instrument*  OR survey*  OR inventory  OR checklist*  )  AND  TS=(  validation  OR validity  OR reliability  OR psychometric*  OR development  OR "content validity"  OR "construct validity"  OR "factor analysis"  OR "internal consistency"  )  AND  TS=(  "assistive technolog*"  OR "rehabilitation technolog*"  OR "training technolog*"  OR "assistive device*"  OR "rehabilitation device*"  OR exoskeleton*  OR "rehabilitation robot*"  OR "assistive robot*"  OR orthos*  OR wheelchair*  OR "prosthetic limb*"  OR "prosthetic device*"  )  NOT  TS=(  dental  OR dentistry  OR orthodont*  OR oral  OR maxillofacial  OR surgical  OR surgery  OR industrial  OR manufacturing  OR army  ) |
| SCOPUS | TITLE-ABS-KEY(  satisfaction  OR "user satisfaction"  OR "patient satisfaction"  OR usability  OR acceptability  OR "user experience"  )  AND  TITLE-ABS-KEY(  questionnaire*  OR scale*  OR instrument*  OR survey*  OR inventory  OR checklist*  )  AND  TITLE-ABS-KEY(  validation  OR validity  OR reliability  OR psychometric*  OR development  OR "content validity"  OR "construct validity"  OR "factor analysis"  OR "internal consistency"  )  AND  TITLE-ABS-KEY(  "assistive technolog*"  OR "rehabilitation technolog*"  OR "training technolog*"  OR "assistive device*"  OR "rehabilitation device*"  OR exoskeleton*  OR "rehabilitation robot*"  OR "assistive robot*"  OR orthos*  OR wheelchair*  OR "prosthetic limb*"  OR "prosthetic device*"  )  AND NOT  TITLE-ABS-KEY(  dental  OR dentistry  OR orthodont*  OR oral  OR maxillofacial  OR surgical  OR surgery  OR industrial  OR manufacturing  OR army  ) |

Table 3 Search strings for conducting the literature review in the PubMed/MEDLINE, Web Of Science and Scopus databases
