# Supplementary material for: User Satisfaction With Assistive, Rehabilitation, and Training Technologies: Questionnaire Development and Content Validation Study
Source: JMIR Rehabil Assist Technol. 2026 Sep 25;13:e88182. doi: 10.2196/88182 (PMC13614623; doi:10.2196/88182)
Supplement: Supplementary file 3 [file rehab-v13-e88182-s003.docx]

**INITIAL SCREENING QUESTIONS**

IQ1: How would you name you assistive/rehabilitation/training technology? (assistive / rehabilitation / training / other)

IQ2: Who is this questionnaire intended for? (Healthcare professional/Trainer / Patient/Participant)

IQ3: In which of the following categories would you put this assistive/rehabilitation/training technology? (assistive / rehabilitation / training)

IQ4: Is this assistive/rehabilitation/training technology wearable? (Yes/No)

IQ5: Is this assistive/rehabilitation/training technology stationary? (Yes/No)

IQ6: Is this assistive/rehabilitation/training technology in contact with the body? (Yes/No)

IQ7: Does this assistive/rehabilitation/training technology use software to operate? (Yes/No)

IQ8: Is there a need to adjust this assistive/rehabilitation/training technology for every user? (Yes/No)

IQ9: Is this assistive/rehabilitation/training technology suited for outside use? (Yes/No)

IQ10: Does this assistive/rehabilitation/training technology need a battery to operate? (Yes/No)

**DESIGN**

D1: I am satisfied with the size of the assistive/rehabilitation/training technology.

D2: I am satisfied with the weight of the assistive/rehabilitation/training technology.

D3: I am satisfied with the ease of transporting the assistive/rehabilitation/training technology.

D4: I am satisfied with the adjustability of the assistive/rehabilitation/training technology to all intended users.

D5: I am satisfied with the adjustability of the assistive/rehabilitation/training technology to my personal needs.

D6: I am satisfied with the ease of adjusting the hardware components (e.g., fastening cuffs, screws, pins) of the assistive/rehabilitation/training technology.

D7: I am satisfied with the ease of using the software of the assistive/rehabilitation/training technology.

D8: I am satisfied with the durability and resistance to wear of the assistive/rehabilitation/training technology.

D9: I am satisfied with the battery life of the assistive/rehabilitation/training technology.

D10: I am satisfied with the ease of using the assistive/rehabilitation/training technology in combination with other everyday items (e.g., shoes with an AFO).

D11: I am satisfied with the ease of cleaning/hygiene of the assistive/rehabilitation/training technology.

D12: I am satisfied with the design and appearance of the assistive/rehabilitation/training technology.

**COMFORT**

C1: I am satisfied with the overall comfort of the assistive/rehabilitation/training technology.

C2: I am satisfied with the comfort of the contact points between my body and the assistive/rehabilitation/training technology.

C3: I am satisfied with the comfort of the contact point/s in the head area.

C4: I am satisfied with the comfort of the contact point/s in the neck area.

C5: I am satisfied with the comfort of the contact point/s in the shoulder area.

C6: I am satisfied with the comfort of the contact point/s in the upper arm area.

C7: I am satisfied with the comfort of the contact point/s in the elbow area.

C8: I am satisfied with the comfort of the contact point/s in the forearm area.

C9: I am satisfied with the comfort level of fixation point/s in the palm/hand area.

C10: I am satisfied with the comfort of the contact point/s in the wrist area.

C11: I am satisfied with the comfort of the contact point/s in the upper back area.

C12: I am satisfied with the comfort of the contact point/s in the lower back area.

C13: I am satisfied with the comfort of the contact point/s in the chest area.

C14: I am satisfied with the comfort of the contact point/s in the abdominal area.

C15: I am satisfied with the comfort of the contact point/s in the hip area.

C16: I am satisfied with the comfort of the contact point/s in the thigh area.

C17: I am satisfied with the comfort of the contact point/s in the knee area.

C18: I am satisfied with the comfort of the contact point/s in the shin area.

C19: I am satisfied with the comfort of the contact point/s in the ankle/foot area.

**EFFECTIVENESS AND USEFULNESS**

EU1: I am satisfied with the number of steps required to accomplish the desired task using the assistive/rehabilitation/training technology.

EU2: I am satisfied with the ability to recover from mistakes while using the assistive/rehabilitation/training technology.

EU3: I am satisfied with the performance of the assistive/rehabilitation/training technology in relation to its intended and expected use.

EU4: I am satisfied with the way the assistive/rehabilitation/training technology enhances my ability to perform daily activities.

**SERVICE**

SE1: I am satisfied with the clarity of the instructions for using the assistive/rehabilitation/training technology.

SE2: I am satisfied with the time needed to understand how the assistive/rehabilitation/training technology is supposed to operate/function.

SE3: I am satisfied with the time needed to become skilful with the assistive/rehabilitation/training technology.

SE4: I am satisfied with the performance of the assistive/rehabilitation/training technology in the specified terrain conditions (e.g., steps, uphill, downhill, flat surface).

**TREATMENT OR EXERCISE**

TE1: I am satisfied with intended frequency of treatment/exercise using the assistive/rehabilitation/training technology.

TE2: I am satisfied with intended content of treatment/exercise using the assistive/rehabilitation/training technology.

TE3: I am satisfied with the recommended duration of treatment/exercise using the assistive/rehabilitation/training technology.

**OVERALL IMPRESSIONS**

OI1: I am satisfied with the assistive/rehabilitation/training technology in general.

OI2: I would recommend the assistive/rehabilitation/training technology to others.

OI3: The assistive/rehabilitation/training technology is pleasant to use.

OI4: I would like to continue using the assistive/rehabilitation/training technology if possible.

OI5: The assistive/rehabilitation/training technology is easy to use.

OI6: I feel that the price of the assistive/rehabilitation/training technology is reasonable considering its benefits and cost per use.

**SAFETY**

SA1: I felt safe and secure when using the assistive/rehabilitation/training technology.

SA2: I have experienced no adverse effects while using the assistive/rehabilitation/training technology.
